# Surface Functionalization of Hydroxyapatite Scaffolds with MgAlEu‐LDH Nanosheets for High‐Performance Bone Regeneration

**DOI:** 10.1002/advs.202204234

**Published:** 2022-11-17

**Authors:** Guanyun Wang, Zehui Lv, Tao Wang, Tingting Hu, Yixin Bian, Yu Yang, Ruizheng Liang, Chaoliang Tan, Xisheng Weng

**Affiliations:** ^1^ Department of Orthopedic Surgery State Key Laboratory of Complex Severe and Rare Diseases Peking Union Medical College Hospital Chinese Academy of Medical Science and Peking Union Medical College Beijing 100730 China; ^2^ State Key Laboratory of Chemical Resource Engineering Beijing Advanced Innovation Center for Soft Matter Science and Engineering Beijing University of Chemical Technology Beijing 100029 P. R. China; ^3^ Department of Chemistry and Center of Super‐Diamond and Advanced Films (COSDAF) City University of Hong Kong Kowloon Hong Kong SAR China; ^4^ Shenzhen Research Institute City University of Hong Kong Shenzhen 518057 P. R. China

**Keywords:** bone repair and regeneration, hydroxyapatite scaffolds, MgAlEu‐layered double hydroxide nanosheets, surface functionalization

## Abstract

Although artificial bone repair scaffolds, such as titanium alloy, bioactive glass, and hydroxyapatite (HAp), have been widely used for treatment of large‐size bone defects or serious bone destruction, they normally exhibit unsatisfied bone repair efficiency because of their weak osteogenic and angiogenesis performance as well as poor cell crawling and adhesion properties. Herein, the surface functionalization of MgAlEu‐layered double hydroxide (MAE‐LDH) nanosheets on porous HAp scaffolds is reported as a simple and effective strategy to prepare HAp/MAE‐LDH scaffolds for enhanced bone regeneration. The surface functionalization of MAE‐LDHs on the porous HAp scaffold can significantly improve its surface roughness, specific surface, and hydrophilicity, thus effectively boosting the cells adhesion and osteogenic differentiation. Importantly, the MAE‐LDHs grown on HAp scaffolds enable the sustained release of Mg^2+^ and Eu^3+^ ions for efficient bone repair and vascular regeneration. In vitro experiments suggest that the HAp/MAE‐LDH scaffold presents much enhanced osteogenesis and angiogenesis properties in comparison with the pristine HAp scaffold. In vivo assays further reveal that the new bone mass and mineral density of HAp/MAE‐LDH scaffold increased by 3.18‐ and 2.21‐fold, respectively, than that of pristine HAp scaffold. The transcriptome sequencing analysis reveals that the HAp/MAE‐LDH scaffold can activate the Wnt/*β*‐catenin signaling pathway to promote the osteogenic and angiogenic abilities.

## Graphical abstract

MgAlEu‐LDH nanosheet‐modified hydroxyapatite scaffolds are designed and fabricated for efficient bone repair and vascular regeneration, which exhibit excellent angiogenic and osteogenic performance due to the improved surface roughness, specific surface, and hydrophilicity as well as the sustained release of Mg^2+^ and Eu^3+^ ions.

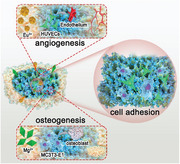

## 1 Introduction

With the rapid aging of the population, bone defects and bone destruction caused by diseases (e.g., osteoporosis, bacterial infections, and bone tumors) or accidental factors (e.g., car accidents and trauma) are becoming increasingly common.^[^ ^1^, ^2^, ^3^ ^]^ Under physiological condition, desired bone defect repair emphasizes the orchestrating of angiogenesis and osteogenesis, of which angiogenesis is the precondition and stimulative for osteogenesis.^[^ ^4^, ^5^ ^]^ However, current angiogenic strategies in bone tissue engineering are mainly stem cell therapy, growth factor therapy, and gene therapy, which are of high cost or raise concerns of tumorigenesis.^[^ ^6^, ^7^, ^8^ ^]^ In view of this, artificial inorganic bone repair materials with intrinsic angiogenic and osteogenic properties as well as good biosafety are promising in reconstructing vascularized bone tissue for bone defect treatment and have been widely studied in recent years.^[^ ^9^, ^10^, ^11^ ^]^ Among them, hydroxyapatite (HAp) is the inorganic component of natural bone tissue containing calcium and phosphorus elements, and has been used as an excellent implant material for bone defect repair.^[^ ^12^ ^]^ HAp scaffold has a hierarchical pore structure that allows deep penetration of blood vessels into the material, thus enabling the reconstruction of internal bone tissue.^[^ ^13^, ^14^, ^15^ ^]^ However, HAp scaffold suffers from some disadvantages such as weak osteoinductive ability, poor cell crawling, and adhesion properties, resulting in the slow bone repair efficiency.^[^ ^16^, ^17^ ^]^ To this end, various methods have been developed to enhance the bioactivity and cell crawling/adhesion properties of HAp scaffolds, such as creating porous surface via 3D printing^[^ ^18^, ^19^, ^20^ ^]^ or surface modification to promote the cell adhesion and differentiation,^[^ ^21^, ^22^, ^23^, ^24^ ^]^ and doping or surface modification with bioactive elements to improve the bioactivity of HAp scaffold.^[^ ^25^, ^26^, ^27^ ^]^ For example, reduced graphene oxide (rGO) has been used to modify the surface of porous HAp to prepare 3D porous HAp/rGO scaffolds with a hierarchical structure.^[^ ^17^ ^]^ The loaded rGO can improve the adhesion and promote the spontaneous osteogenic differentiation and proliferation of bone marrow mesenchymal stem cells (BMSCs). In addition, a robust biosilicification strategy has been developed to impart a uniform and stable osteoinductive surface to porous collagen scaffolds, resulting in a native‐bone‐like porous structure and a nanosilica coating.^[^ ^28^ ^]^ It was found that the osteoinductivity of nanosilica‐collagen scaffolds are dependent on the surface roughness and silicon content in the silica coating. However, those reported strategies can only achieve either enhanced bioactivity or cell crawling/adhesion properties in previous studies.^[^ ^29^, ^30^, ^31^, ^32^, ^33^, ^34^ ^]^ Therefore, it still remains challenging to develop simple but effective strategies for preparation of HAp‐based scaffolds with excellent osteogenic/angiogenesis performance and cell crawling and adhesion properties.

2D layered double hydroxides (LDHs) have been widely explored in biomedical fields such as tumor diagnosis, cancer therapy, and drug delivery in last decade.^[^ ^35^, ^36^, ^37^, ^38^ ^]^ The excellent biocompatibility, high specific surface area, and tunable chemical composition of 2D LDHs also make them promising bioactive materials for bone tissue engineering.^[^ ^39^, ^40^, ^41^, ^42^, ^43^ ^]^ For example, Mg‐based LDH has shown excellent osteogenesis performance compared with pure Mg and Mg alloy.^[^ ^44^, ^45^, ^46^, ^47^ ^]^ In our previous studies, Yb‐containing MgAl‐LDH nanosheets loaded with alendronate exhibited excellent osteogenic differentiation and bone regeneration properties in vitro and in vivo.^[^ ^48^ ^]^ MgAl‐LDH microsheet‐incorporated polymethyl methacrylate (PMMA) bone cements could promote bone formation in vivo and improve interfacial osseointegration between bone and bone cement.^[^ ^49^ ^]^ In addition, rare earth ions such as gadolinium (Gd), europium (Eu), cerium (Ce), and ytterbium (Yb), have unique advantages in bone repair. For example, Eu^3+^ has been proved to possess favorable angiogenic properties and can up‐regulate the expression of angiogenic genes, while Ce^3+^ can reduce potential oxidative stress by inhibiting the expression of inflammation‐related genes.^[^ ^50^, ^51^, ^52^, ^53^, ^54^, ^55^, ^56^, ^57^ ^]^ Therefore, it is expected that surface functionalization of rare earth‐containing Mg‐based LDH nanosheets on porous HAp scaffold will not only endow the HAp scaffold with excellent osteogenic and angiogenic properties, but also improve the roughness of the scaffold surface and thus provide more adsorption sites for cell adhesion and differentiation.

**Scheme 1:**
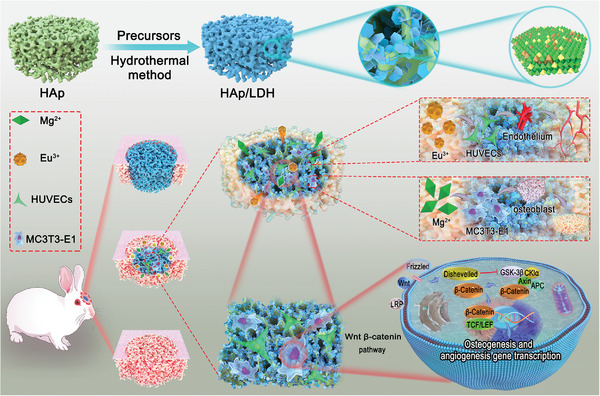
Schematic illustration of the fabrication process and the excellent angiogenic and osteogenic properties as well as corresponding underlying mechanism of the MAE‐LDH nanosheet‐modified hydroxyapatite scaffold.

In this contribution, we report a simple but effective surface functionalization strategy to prepare HAp/MAE‐LDH scaffolds through the in situ growth of MgAlEu‐LDH (MAE‐LDH) nanosheets on porous HAp scaffolds for enhanced bone regeneration (**Scheme** **1**). The surface roughness, specific surface and hydrophilicity of the HAp scaffold are significantly enhanced after surface functionalization with MAE‐LDHs, resulting in the effective enhancement on its cells adhesion and osteogenic differentiation (Scheme 1). Moreover, the MAE‐LDHs functionalized on HAp scaffolds enable the sustained release of Mg^2+^ and Eu^3+^ ions for efficient bone repair and vascular regeneration (Scheme 1). Importantly, in vitro experiments suggest that the HAp/MAE‐LDH scaffold presents much enhanced osteogenesis and angiogenesis properties in comparison with the pristine HAp scaffold. In vivo assays further reveal that the new bone mass and mineral density of HAp/MAE‐LDH scaffold increased by 3.18‐ and 2.21‐fold respectively than that of pristine HAp scaffold. The transcriptome sequencing analysis reveals that the HAp/MAE‐LDH scaffold could activate the Wnt/*β*‐catenin signaling pathway to promote the osteogenic and angiogenic abilities (Scheme 1). Our study provides a simple but effective surface functionalization strategy to fabricate LDH‐functionalized HAp scaffolds to realize much enhanced bone regeneration, which may have the great potential application in practical bone repair and regeneration.

## 2 Results and Discussion

### 2.1 Fabrication and Morphology of HAp/MAE‐LDH Scaffold

In a typical process, pure HAp scaffolds were first prepared by a soft‐template method according to the previous literature.^[^ ^17^ ^]^ Subsequently, MAE‐LDH nanosheets were grown on the surface of the HAp scaffold by the hydrothermal method (Figure S1 and see details in the Experimental Section in the Supporting Information). The shrinkage rate during the preparation of HAp scaffolds was determined to be 40 ± 3.5%. As shown in Figure S2 (Supporting Information), the scanning electron microscopy (SEM) images show that both HAp and HAp/MAE‐LDH scaffolds exhibit highly porous microstructures with pore sizes ranging from 300 to 500 µm. Such a pore size is favorable for desirable cell infiltration and growth inside the scaffolds for bone tissue engineering.^[^ ^58^, ^59^ ^]^ The pore surface of the HAp scaffold is smooth, while the HAp/MAE‐LDH scaffold displays an interconnected rough surface grown with flake‐like vertical MAE‐LDH nanosheet network, indicating the successful growth of MAE‐LDHs on the HAp scaffold.

**Figure 1:**
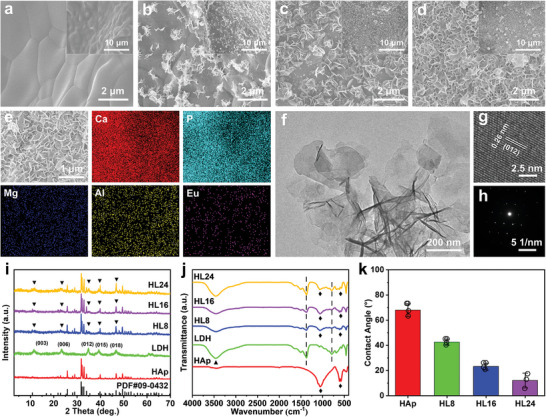
SEM images of a) HAp, b) HL8, c) HL16, and d) HL24 scaffolds. e) EDX mapping of HL24 scaffold. f) TEM and g,h) HR‐TEM images of MAE‐LDHs. i) XRD patterns of HAp, MAE‐LDHs, HL8, HL16, and HL24 scaffolds with inserted reference. j) FT‐IR spectra of HAp, MAE‐LDHs, HL8, HL16, and HL24 scaffolds. k) Contact angles of HAp, HL8, HL16, and HL24 scaffolds. Data are presented as mean values ± s.d. (*n* = 3).

The effect of different hydrothermal treatment times (8, 16, 24, 36, and 48 h) on the MAE‐LDHs grown on HAp scaffold surface (denoted as HL8, HL16, HL24, HL36, and HL48) was investigated. As shown in **Figure** **1**a–d, the density of MAE‐LDHs grew on the scaffold surface increases as the hydrothermal reaction time increases. It should be pointed out that HL36 and HL48 have the same surface morphology as HL24 (Figure S3a,b, Supporting Information), indicating that the growth of MAE‐LDHs saturates at 24 h. The elemental contents of these HAp/MAE‐LDH scaffolds measured by inductively coupled plasma (ICP) emission spectrometry verified the aforementioned results, since the concentrations of Mg, Al, and Eu elements in HAp/MAE‐LDH increased gradually with hydrothermal reaction time and reached the maximum at 24 h (Figure S3c, Supporting Information). HL24 scaffolds with different Eu doping contents (HL24‐Eu/10%, HL24‐Eu/20%, and HL24‐Eu/30%) were also prepared, and ICP analysis showed no significant difference in their Mg, Al, and Eu element contents (Figure S3d, Supporting Information). The growth of MAE‐LDHs on the surface of the HAp scaffold was further verified by energy dispersive X‐ray spectroscopy (EDX) elemental mapping. A homogeneous distribution of Mg, Al, and Eu elements of MAE‐LDHs was clearly observed in addition to Ca and P elements of the HAp scaffold (Figure 1e). According to transmission electron microscopy (TEM) image, the MAE‐LDHs have a plate‐like nanosheet structure with a lateral size of 300–500 nm (Figure 1f), which is consistent with the SEM results. High‐resolution TEM (HR‐TEM) image showed the good crystallinity of MAE‐LDHs with ordered lattice fringes and an interplanar spacing of 0.26 nm, which is attributed to the (012) plane of the LDH crystal (Figure 1g). The selected area electron diffraction (SAED) pattern further reveals the single‐crystalline nature of MAE‐LDH nanosheets (Figure 1h)

### 2.2 Characterization of Different HAp/MAE‐LDH Samples

X‐ray diffraction (XRD) pattern was employed to characterize the crystal structure of different HAp/MAE‐LDH samples (HL8, HL16, and HL24). As shown in Figure 1i, the diffraction peaks of the as‐prepared HAp scaffolds match well with the standard pattern of the HAp crystal phase (PDF#09‐0432). The presence of MAE‐LDHs in the HAp/MAE‐LDH scaffolds was evident by the XRD characterization. A series of characteristic peaks corresponding to (003), (006), (012), (015), and (018) reflections of LDHs were found in the patterns of HL8, HL16, and HL24 scaffolds (Figure 1i). The growth of MAE‐LDHs on HAp scaffold was also confirmed by Fourier transform infrared (FT‐IR) spectroscopy. As shown in Figure 1j, the characteristic bands at 3420 cm^−1^ (stretching vibration of hydroxyl groups), 1359 cm^−1^ (asymmetric stretching vibration of C=O in CO_3_^2−^), and 445 cm^−1^ (bending vibration of O–M–O) of MAE‐LDHs were observed in the HL8, HL16, and HL24 samples, accompanied with the absorption bands of the HAp scaffold at 1037 cm^−1^ (asymmetric stretching vibration of O–P) and 607 cm^−1^ (bending vibration of O–P–O), indicating the coexistence of MAE‐LDHs and HAp scaffold.

X‐ray photoelectron spectroscopy (XPS) was performed to investigate the chemical compositions and their valence states of the HAp/MAE‐LDH scaffold. Compared with the pure HAp scaffold (Figure S4a, Supporting Information), peaks originating from Mg, Al, and Eu were found in the survey spectrum of the HL24 scaffold (Figure S4b, Supporting Information), which is consistent with the EDX results in Figure 1e. The XPS Mg 2p and Al 2p spectra of the HL24 scaffold in Figure S4c (Supporting Information) revealed the presence of Mg^2+^ (binding energy (BE) at 50.1 eV) and Al^3+^ species (BE at 74.1 eV). The high‐resolution XPS Eu 3d spectra (Figure S4d, Supporting Information) showed two main peaks at 1135.3 eV (Eu 3d_5/2_) and 1165.1 eV (Eu 3d_3/2_), demonstrating the existence of Eu^3+^ species in the HL24 scaffold.

Given that the specific hierarchical pores of the scaffolds are critical for cell infiltration, osteogenesis, and blood vessel in‐growth, the porosity of each HAp/MAE‐LDH scaffold was characterized. The porosity of pure HAp scaffold is 76.96 ± 0.90% (Figure S5a, Supporting Information), and the HL8, HL16 and HL24 scaffolds exhibit similar results with the porosity of 76.50 ± 0.70%, 76.08 ± 1.16%, and 75.42 ± 0.62%, respectively, demonstrating that the growth of MAE‐LDH nanosheets has little effect on the porous structure of the HAp scaffold. The specific surface area (SSA) of each HAp/MAE‐LDH scaffold was also investigated. It was found that the SSA of the HAp/MAE‐LDH scaffolds increases with the prolongation of hydrothermal treatment time, and the HL24 scaffold displays the highest SSA (Figure S5b, Supporting Information), which is conducive to providing more sites for cells adhesion to promote their proliferation and differentiation. The hydrophilicity of each HAp/MAE‐LDH scaffold was estimated by measuring the contact angles. Figure 1k showed that all HAp/MAE‐LDH scaffolds exhibit smaller contact angles compared to the pure HAp scaffold, indicating the improved hydrophilicity of these scaffolds after growth of MAE‐LDHs on their surface. Among them, the HL24 scaffold has the best hydrophilicity with the smallest contact angle of 12.6 ± 4.9°, which would facilitate cell attachment. In addition, the HL8, HL16, and HL24 scaffolds exhibited various ions release kinetic behaviors over 35 d (Figure S6, Supporting Information), and the release amounts were far below the cytotoxic level.^[^ ^60^ ^]^ As expected, these HAp/MAE‐LDH scaffolds could achieve sustainable release of bioactive ions, which are necessary for promoting cell adhesion, osteogenesis, and angiogenesis.

### 2.3 Biocompatibility of HAp/MAE‐LDH Scaffolds

To assess the biocompatibility of HAp/MAE‐LDH scaffolds, cell‐counting kit‐8 (CCK‐8) assay and Live/Dead fluorescence staining were performed on MC3T3‐E1 and Human Umbilical Vein Endothelial Cells (HUVECs). As shown in Figure S7 (Supporting Information), similar cell numbers and optical density (OD) values of MC3T3‐E1 were observed in the pristine HAp group and HAp/MAE‐LDH groups on days 1, 3, 5, and 7, respectively, demonstrating their excellent biocompatibility. It was found that much more HUVECs and higher OD value of HUVECs were observed in HL24 group on days 1, 3, 5, and 7 compared with the HAp group, indicating that the introduced LDHs possess the ability to promote the proliferation of HUVECs (Figure S8, Supporting Information). The adhesion performance of MC3T3‐E1 cells on each HAp/MAE‐LDH scaffold was evaluated by SEM after culturing for 3 d. As shown in Figure S9 (Supporting Information), MC3T3‐E1 cells on all HAp/MAE‐LDH scaffolds exhibit extended and flat filopodia as compared to that on the HAp scaffold. Moreover, MC3T3‐E1 cell on the HL24 scaffold spread more fully, which could be attributed to its optimal specific surface area and hydrophilicity.

### 2.4 In Vitro Osteogenic Properties of HAp/MAE‐LDH Scaffolds

**Figure 2:**
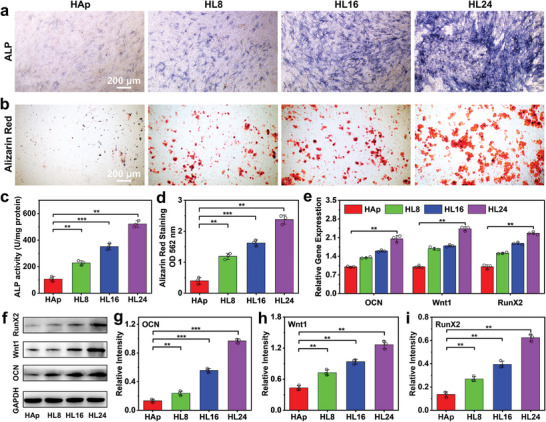
The osteogenic properties of HAp/MAE‐LDH scaffolds. a,b) Optical microscope images of Alkaline phosphatase staining and Alizarin red S staining of MC3T3‐E1 cultured with HAp, HL8, HL16, and HL24 scaffolds. Scale bar: 200 µm. c,d) Quantitative analysis of Alkaline phosphatase staining and Alizarin red S staining using ImageJ 1.52q1.52v software. e) qPCR results detecting the osteogenic gene (OCN, Wnt1, and RunX2) expression of MC3T3‐E1 cultured with HAp, HL8, HL16, and HL24 scaffolds. f) Western blot results detecting the osteogenic proteins (OCN, Wnt1, and RunX2) expression of MC3T3‐E1 cultured with HAp, HL8, HL16, and HL24 scaffolds. g–i) Quantitative analysis of the Western blot results evaluating osteogenic proteins expression of MC3T3‐E1 using ImageJ 1.52q1.52v software. Data are presented as mean values ± s.d. (*n* = 3). **p < 0.01, ***p < 0.001 (one‐way ANOVA).

The alkaline phosphatase (ALP) and alizarin red S staining assays were performed on MC3T3‐E1 to evaluate the in vitro osteogenic properties of HAp/MAE‐LDH scaffolds. As shown in **Figure** **2**a,b, significantly more pronounced coloration in ALP as well as apparent mineralized nodules in alizarin red S staining were observed in HL24 group compared with other groups after 14‐d culture. Further quantitative results presented in Figure 2c,d confirmed the intuitive results, where 4.89‐fold of ALP activity and 5.96‐fold of alizarin red S OD values were observed in HL24 group compared with that in the HAp group. Moreover, quantitative polymerase chain reaction (qPCR) experiments and western blotting assays were carried out to investigate the transcription and translation levels of osteogenic‐related gene expression. In Figure 2e–i, osteogenic marker genes and proteins expressed during osteogenic differentiation, including osteocalcin (OCN), Wnt1, and Runt‐related transcription factor 2 (Runx2), were detected in pristine HAp group and HAp/MAE‐LDH groups. Notably, much higher osteogenic maker genes (2.05‐fold for OCN, 2.43‐fold for Wnt1, and 2.26‐fold for Runx2) and proteins (7.35‐fold for OCN, 2.93‐fold for Wnt1, and 4.59‐fold for Runx2) expression were observed in the HL24 group compared with that in HAp group, indicating that the MAE‐LDHs possess remarkable osteogenic properties and can upregulate the expression of osteogenic genes and proteins.

### 2.5 In Vitro Angiogenic Properties of HAp/MAE‐LDH Scaffolds

**Figure 3:**
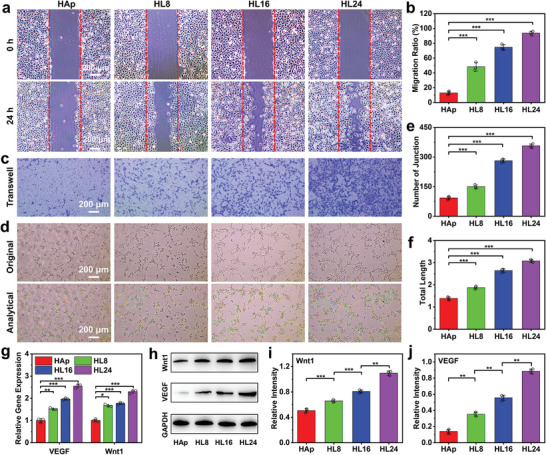
a,b) Optical microscope images and further quantitative analysis results (ImageJ 1.52q1.52v software) of scratch assay evaluating the migration activity of HUVECs cultured with HAp, HL8, HL16, and HL24 scaffolds. c) Migrated HUVECs cultured with HAp, HL8, HL16, and HL24 scaffolds stained with crystal violet in Transwell assay. d) Matrigel experiment results accessing the vessel generation capability of HUVECs cultured with HAp, HL8, HL16, and HL24 scaffolds. e,f) Quantitative analysis of the Matrigel experiment results counting the number of joint and total length formed by HUVECs using ImageJ 1.52q1.52v software. g) qPCR results of the angiogenic gene (VEGF and Wnt1) expression of HUVECs cultured with HAp, HL8, HL16, and HL24 scaffolds. h) Western blot results of the angiogenic protein (VEGF and Wnt1) expression of HUVECs cultured with HAp, HL8, HL16, and HL24 scaffolds. i,j) Quantitative analysis of the Western blot results evaluating the angiogenic protein expression of HUVECs using ImageJ 1.52q1.52v software. Data are presented as mean values ± s.d. (*n* = 3). *p < 0.05, **p < 0.01, ***p < 0.001 (one‐way ANOVA).

Scratch assay, transwell assay, and tube formation assay were conducted on HUVECs to evaluate the angiogenic properties of HAp/MAE‐LDH scaffolds. As shown in **Figure** **3**a,b, an increased migration ratio of HUVECs was found in scaffolds with higher content of LDHs, and the 7.24‐fold migration ratio of HUVECs was observed in the HL24 group compared with that in HAp group. The similar results were confirmed in transwell assay, where enhanced migration activity of HUVECs was observed in scaffolds with higher content of LDHs (Figure 3c). Moreover, tube formation assay showed that the length of tube and number of junctions were significantly increased in scaffolds with higher content of LDHs (Figure 3d; Figure S10, Supporting Information). Further quantitative analysis demonstrated that the junction number and total length in HL24 group were 3.86‐ and 2.21‐fold higher than those in HAp group (Figure 3e,f). Furthermore, scratch assay, transwell assay, and tube formation assay were also performed on HUVECs cultured with HAp/MgAl‐LDH or HAp/MAE‐LDH, where significantly more potent angiogenic activities were observed in the HAp/MAE‐LDH group compared with the HAp/MgAl‐LDH group (Figures S11––S13, Supporting Information), demonstrating the enhanced angiogenic properties of HAp/MAE‐LDH derived from released Eu^3+^. Besides, angiogenesis‐related genes including Wnt1 and VEGF were detected to further verify the angiogenic properties of HAp/MAE‐LDH scaffolds. As shown in Figure 3g, higher angiogenic gene expression was found in scaffolds with higher LDHs content, and the transcription levels of Wnt1 and VEGF were most significantly upregulated in HL24 group, which is attributed to the sustained release of Eu^3+^ from the MAE‐LDHs. Similar results were also obtained in western blotting by detecting the expression of the angiogenic‐related protein, indicating the excellent angiogenic properties of MAE‐LDHs (Figure 3h–j).

### 2.6 The Underlying Mechanism of the Osteogenic and Angiogenic Properties of HAp/MAE‐LDH Scaffolds

**Figure 4:**
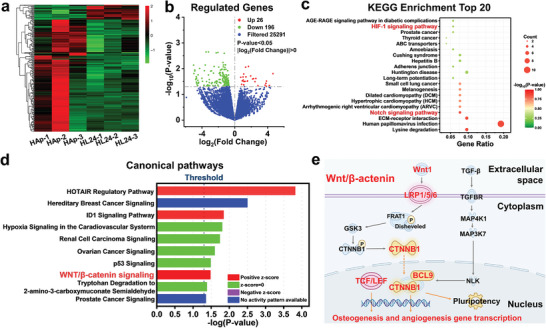
a,b) Significantly differentially expressed genes identified by transcriptome sequencing are shown in the heatmap and volcano plot, the activated genes are marked in red and the silenced genes are marked in green. Cutoff: *P* value < 0.05 and |log2 FC| > 1. c) The top 20 signal pathways enriched by differentially expressed genes identified by KEGG analysis. d) Ingenuity Pathway Analysis revealed the potential involved osteogenic and angiogenic pathways. e) The differentially expressed genes clustered in wnt/*β*‐catenin signaling pathway based on the transcriptome sequencing and IPA analysis.

To further explore the underlying mechanism of the osteogenic and angiogenic properties of HAp/MAE‐LDH scaffolds, transcriptome sequencing was performed using MC3T3‐E1 cultured with pristine HAp or HL24 scaffold. The heatmap revealed the differentially expressed genes of MC3T3‐E1, where up‐regulated genes are marked in red while down‐regulated genes are marked in green (**Figure** **4**a). Specifically, 222 differentially expressed genes (*P* value < 0.05 & |log2 Fold Change| > 1) were identified, among which 26 genes are up‐regulated and 196 genes are down‐regulated (Figure 4b). Based on the numerous differentially expressed genes, the admirable osteogenic and angiogenic properties of HL24 scaffold were verified in the above experiments. Kyoto encyclopedia of genes and genomes (KEGG) analysis was further performed to explore the potential signal pathways involved in the osteogenic and angiogenic processes. As shown in Figure 4c, the top 20 related signal pathways were identified, among which the HIF1 signaling pathway and Notch signaling pathway are considered to play important roles in the augmentation of osteogenesis and angiogenesis. Moreover, ingenuity pathway analysis (IPA, v01‐04, QIANGEN Redwood City, CA, USA) software based on specific algorithms was utilized to further identify the potential osteogenic and angiogenic pathways. It turned out that wnt/*β*‐catenin signaling was significantly activated during the process (Figure 4d), which is known to play an essential role in regulating cell proliferation, vascular plexus remodeling, and bone regeneration.^[^ ^61^ ^]^ Thus, differentially expressed genes regulated by HAp/MAE‐LDH in the wnt/*β*‐catenin signaling pathway are enriched and marked in orange or blue to visually delineate the transcriptome sequencing results, where the orange represents activated genes and blue represents inhibited genes, and the shade of color indicates the degree of activation or inhibition (Figure 4e; Figure S14, Supporting Information).

### 2.7 In Vivo Osteogenic and Angiogenic Properties of HAp/MAE‐LDH Scaffolds

**Figure 5:**
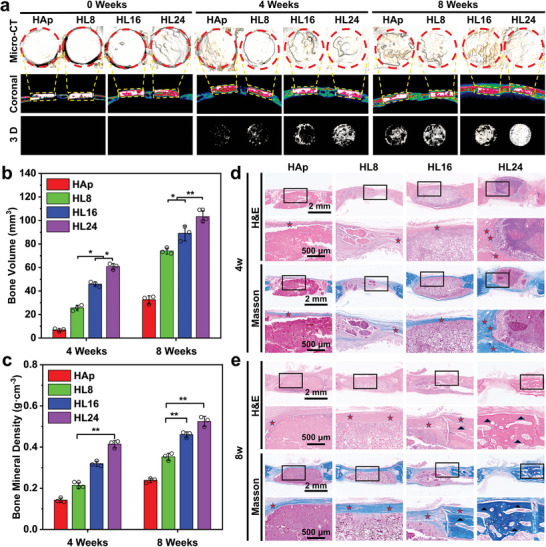
a) Newly formed bone tissue induced by HAp, HL8, HL16, and HL24 scaffolds implantation detected by horizontal, coronal, and 3D reconstructed Micro‐computed tomography (Micro‐CT) scanning. b,c) Quantitative morphometric analysis of the Micro‐CT results counting the bone volume and bone mineral density of regenerated bone tissue. d,e) Newly formed bone tissue induced by HAp, HL8, HL16, and HL24 scaffolds implantation stained with Hematoxylin and eosin (H&E) staining and Masson trichrome staining, the regenerating bone tissue is shown at low and high magnification. Data are presented as mean values ± s.d. (*n* = 3). *p < 0.05, **p < 0.01 (one‐way ANOVA).

Critical‐size calvarial defect models of New Zealand rabbits are utilized to evaluate the in vivo osteogenic properties of HAp/MAE‐LDH scaffolds. Specifically, after the calvarial defect model were built, the scaffolds were implanted into the corresponding positions (Figure S15, Supporting Information) and allowed for reconstructing surrounding bone structure for 4 or 8 weeks. The horizontal, coronal, and 3D reconstructed micro‐computed tomography (Micro‐CT) scanning images were taken at 0, 4, and 8 weeks postoperatively. As shown in **Figure** **5**a, significantly enhanced new bone formation was observed in HAp/MAE‐LDH compared with pristine HAp at both 4 and 8 weeks, and the highest new bone volume (BV) was found in HL24 group. Similar results were obtained in further quantitative analysis of these Micro‐CT images, where 3.18‐fold BV and 2.21‐fold bone mineral density (BMD) were observed in HAp/MAE‐LDH groups compared with that in HAp group (Figure 5b,c). Moreover, histological staining including hematoxylin and eosin (H&E) and Masson's trichrome staining were also performed to verify the enhanced bone regeneration properties of HAp/MAE‐LDH scaffolds. Consistent with the results of Micro‐CT, the H&E staining revealed amounts of regenerated bone and blood vessels in the defect center in the HAp/MAE‐LDH groups at both 4 and 8 weeks postoperatively, while only a little neo‐bone formation was found in the defect margin in the HAp group (Figure 5d). Masson's trichrome staining also demonstrated an obvious osteoid matrix regenerated within the defect areas, with a larger amount of osteoid and mature bone/mineralized bone (blue color in Masson's trichrome staining) formation observed along the host bone border 8 weeks after the implantation of HAp/MAE‐LDH scaffolds (Figure 5e). Moreover, amounts of small clusters of mature bone were observed in the defect center in HAp/MAE‐LDH groups, as revealed in both H&E staining and Masson's trichrome staining (Figure 5d,e), indicating the superb osteogenic characteristics of HAp/MAE‐LDH. Additionally, the fluorescence intensity of CD31 indicating vascular network maturity was detected. As shown in Figure S16 (Supporting Information), significantly higher CD31 expression intensity was observed in HL8, HL16, and HL24 groups than in the control group, and the CD31 intensity gradually enhanced with the increase of MAE‐LDH components, indicating the favorable in vivo angiogenesis property of HAp/MAE‐LDH scaffolds. The chicken chorioallantoic membrane (CAM) assay was also performed to verify the in vivo angiogenic properties of the HAp/MAE‐LDH scaffolds. Significant more neovascularization was observed in HL24 group than the other groups in the gross photographs, which was further verified by subsequent quantitative analyses after 5‐d coincubation of the CAM and the scaffolds, indicating the favorable in vivo angiogenesis property of HAp/MAE‐LDH scaffolds (Figure S17, Supporting Information). In addition, the in vivo biocompatibility of HL24 scaffold was evaluated by H&E staining of heart, liver, spleen, lung, and kidney tissue sections after scaffold implantation for 8 weeks. As presented in Figure S18 (Supporting Information), no difference was found in any organ tissue sections between week 0, week 4, and week 8, demonstrating negligible systemic toxicity of the HAp/MAE‐LDH scaffold.

## 3 Conclusion

In conclusion, we have developed a simple but effective strategy to fabricate HAp/MAE‐LDH scaffolds with much enhanced osteogenic and angiogenic properties by in situ growth of MAE‐LDHs on the surface of porous HAp scaffolds for highly efficient bone regeneration. The 3D network structure of MAE‐LDH nanosheets on HAp scaffold can significantly improve its roughness, specific surface, and hydrophilicity, resulting in enhanced cell attachment and osteogenic differentiation performance. Moreover, the HAp/MAE‐LDH scaffold possesses the sustained release of Mg^2+^ and Eu^3+^ ions for 35 d, and the release of Mg^2+^ and Eu^3+^ ions further promote the osteogenesis and angiogenesis performance. In vitro experiments demonstrated the obviously improved osteogenesis and angiogenesis properties of HAp/MAE‐LDH scaffold in compared with the pristine HAp scaffold. In vivo experiments also indicated that the new bone mass and bone mineral density in calvarial defect model treated with HAp/MAE‐LDH scaffold was 3.18‐ and 2.21‐folds than that of pure HAp scaffold, respectively at the eighth week postoperatively. This strategy might be a universal method to further extend to functionalize other artificial bone repair scaffolds such as magnesium alloy, stainless steel and titanium alloy, yielding much enhanced bone repair and regeneration performance. Such a simple but effective strategy may have the great potential to realize much enhanced performance on artificial bone repair scaffolds in real clinical application.

## Conflict of Interest

The authors declare no conflict of interest.

## Supporting information

Supporting Information

## Data Availability

The data that support the findings of this study are available from the corresponding author upon reasonable request.
