# Supplementary material for: Surface Functionalization of Hydroxyapatite Scaffolds with MgAlEu‐LDH Nanosheets for High‐Performance Bone Regeneration
Source: Adv Sci (Weinh). 2022 Nov 17;10(1):2204234. doi: 10.1002/advs.202204234 (PMC9811441; doi:10.1002/advs.202204234)
Supplement: Supplementary file 1 — Supporting Information [file ADVS-10-2204234-s001.pdf]

Supporting Information

Surface Functionalization of Hydroxyapatite Scaffolds with MgAlEu-LDH Nanosheets for High-Performance Bone Regeneration

Guanyun Wang,[†] *Zehui Lv,*[†] *Tao Wang,*[†] *Tingting Hu, Yixin Bian, Yu Yang, Ruizheng Liang,* Chaoliang Tan,* and Xisheng Weng**

[†]These authors contributed equally: Guanyun Wang, Zehui Lv, Tao Wang.

Experimental Section

***Chemicals*:** Magnesium nitrate hexahydrate ($Mg(NO_3)_2·6H_2O$, 99.0%), aluminum nitrate nonahydrate ($Al(NO_3)_3·9H_2O$, 99.0%), europium nitrate hexahydrate ($Eu(NO_3)_3·6H_2O$, 99.9%), hexamethylenetetramine ($C_6H_{12}N_4$, ≥ 99.0%) were acquired from Aladdin Biochemical Technology Co., Ltd (Shanghai, China). Hydroxyapatite (HAp) nanopowder (purity ≥ 97%) was obtained from Macklin Biochemical Co., Ltd (Shanghai, China). Chitosan (CS) with an acetylation degree of 90% and a viscosity of 20-1000 MPa·s was acquired from TCI Development Co., Ltd (Shanghai, China). The melamine foam was obtained from Beiyou Building Materials Co., Ltd. (Shanghai, China).

***Preparation of HAp scaffold*:** HAp scaffold was synthesized by a soft template method according to the previous literature.[1] Firstly, to prepare the homogeneous HAp/CS composite slurry, 0.3 g CS powder was added into the aqueous acetic acid solution (200 μL acetic acid was added into 10 mL of deionized (DI) water, and the mixture was stirred magnetically for 2 h until CS powder was completely dissolved). Then, the HAp nanopowder (6 g) was added slowly to CS solution and kept mechanical stirring for another 2 h to obtain homogeneous HAp/CS composite slurry. Secondly, melamine sponges of different shapes were immersed into the HAp/CS composite slurry until the sponges were filled with the slurry. Then, the sponges were transferred to a vacuum oven and kept at 60 °C for 24 h with the vacuum degree of 80 kPa. During vacuum drying, the dry porous nano-HAp/CS/foam scaffolds were obtained as the solvent evaporated. Finally, the pure HAp scaffolds were obtained by calcination at 1350 °C for 2 h at a heating rate of 5 °C/min in a muffle furnace.

***Preparation of HAp/MAE-LDH scaffolds*:** HAp/MAE-LDH scaffolds were fabricated by growth of MAE-LDH nanosheets on HAp scaffold substrates *via* a simple hydrothermal method. For comparison, HAp/MAE-LDH samples with different hydrothermal reaction times were prepared and marked as HL8 (HAp/MAE-LDH 8 h), HL16 (HAp/MAE-LDH 16 h), HL24 (HAp/MAE-LDH 24 h), HL36 (HAp/MAE-LDH 36 h) and HL48 (HAp/MAE-LDH 48 h), respectively. Briefly, HAp scaffolds were carefully cleaned with DI water and absolute ethanol in

an ultrasound bath for 5 min each time. $Mg(NO_3)_2·6H_2O$ (1.2 mmol), $Al(NO_3)_3·9H_2O$ (0.54 mmol), $Eu(NO_3)_3·6H_2O$ (0.06 mmol), and hexamethylenetetramine (6 mmol) were dissolved into DI water (20 mL) and stirred at room temperature to form a clear solution. The above solution and 2.0 g HAp scaffolds were transferred into a Teflon-lined stainless-steel autoclave (25 mL) and maintained at 120 °C for 8 h, 16 h, 24 h, 36 h and 48 h, respectively. The resulting HL8, HL16, HL24, HL36 and HL48 scaffolds samples were rinsed with DI water and absolute ethanol for three times, respectively. After dialysis (3.5 kDa) treatment, the remaining hexamethylenetetramine in the HL8, HL16, HL24, HL36 and HL48 scaffolds were removed. Finally, the HL8, HL16, HL24, HL36 and HL48 scaffolds were transferred to a vacuum oven and dried at 60 °C for 12 h. The MAE-LDH nanosheets were synthesized by the same method without HAp scaffold and the hydrothermal reaction time was 24 h. HL24 scaffolds with different Eu content (HL24-Eu/10%, HL24-Eu/20% and HL24-Eu/30%) were also prepared by the above method except for changing the Eu: Al ratios to 10%, 20%, and 30%, respectively.

Materials characterization**:** The morphology of scaffold samples was examined with a Zeiss SUPRA 55 field-emission scanning electronic microscope (FESEM, Zeiss, Germany) at an accelerating voltage of 20 kV. A Hitachi HT7700 transmission electron microscope (TEM) and a JEOL high-resolution transmission electron microscope (HRTEM, JEOL Ltd., Tokyo, Japan) were used to characterize the morphology of LDHs with an accelerating voltage of 200 kV. The phase compositions of the samples were determined by a Shimadzu XRD-6000 diffractometer (XRD, Shimadzu Corp., Kyoto, Japan) using the Cu Kα radiation at 40 kV and 30 mA current. Fourier transform infrared spectra were recorded by a VERTEX 70v (FT-IR, Bruker Corporation, Germany) spectrophotometer from the range of 400 to 4000 cm^{-1} with 2 cm^{-1} resolution. The surface chemical composition and elemental valence states of HAp and HL24 scaffolds were determined by an X-ray photoelectron spectroscope (XPS, AXIS Supra, Shimadzu, Japan).

The porosity of scaffold samples was measured with the absolute ethanol displacement technique according to previous reports.[2] The specific surface area

(SSA) of the difference scaffold samples were obtained by analyzing N_2 adsorption/desorption through a Brunner-Emmet-Teller measurement (BET, ASAP-2460-4N, Micromeritics, USA). The surface hydrophilicity of each sample was evaluated by measuring the contact angle using a contact angle goniometer (K100, Kruss, Germany). Inductively coupled plasma emission spectroscopy (ICPS-7500, Shimadzu, Japan) was performed to determine the ion content in each scaffold after hydrothermal reaction, and the ion release of samples in phosphate-buffered saline (PBS) solution was performed at pH 7.4 for 1, 3, 5, 7, 14, 21, 28, and 35 days in a shaking water bath at 120 rpm (37 °C). The ratio of PBS solution volume to sample mass was 200 mL g^{-1}. All experiments were performed in triplicate.

***Cell adhesion on scaffolds in vitro*:** MC3T3-E1 mouse pre-osteoblasts (MC3T3-E1 cells) were purchased from Peking Union Medical College Hospital and approved by the hospital ethics committee for in vitro studies. The scaffolds were sterilized with autoclave and gently placed into 48-well plates, where cells were seeded in the scaffolds with a density of 0.5×10^4 per well. Then, the cells were fixed with 2.5% glutaraldehyde and dehydrated with graded ethanol (10%, 30%, 50%, 70%, 80%, 90%, and 100% v/v) and hexamethyldisilazane (Macklin Biochemical Co., Ltd) for the subsequent SEM observation *via* a Zeiss SUPRA 55 scanning electron microscope (Zeiss, Germany).

***In vitro biocompatibility evaluation*:** Sterilized HAp, HL8, HL16, and HL24 scaffolds were fabricated into 6 mm diameter and 2.5 mm thickness disc-shaped specimens for the following experiments. MC3T3-E1 and HUVECs were used to study the biocompatibility of HAp, HL8, HL16, and HL24 scaffolds. Briefly, 10,000 cells seeded in the lower chamber of a 24-well transwell plate with a 3 μm pore diameter (Corning, #3415, USA) were co-cultured with a specimen that was placed in the upper chamber of the transwell plate for 12 h. CCK-8 assay and Live/Dead Cell Staining were performed on days 1, 3, and 5 according to the kit instructions. A multifunctional microplate reader (Varioskan Flash; Thermo Fisher Scientific, USA) was used to record the OD values at 450 ± 5 nm and a confocal laser microscope (LSCM, LSM800, Zeiss, Germany) was used to observe the Live/Dead Cell Staining

results.

In vitro osteogenic properties evaluation**:** To assess the in vitro osteogenic properties of HAp, HL8, HL16, and HL24 scaffolds, MC3T3-E1 were cultured in the same way as described in the section of in vitro biocompatibility evaluation. On Day 14, the differentiated MC3T3-E1 were immersed with 4% paraformaldehyde (Gibco, USA) for 30 min and stained by alkaline phosphatase staining solution (Solarbio Life Science, Beijing, China) or alizarin red S solution (Solarbio Life Science, Beijing, China) for another 30 min. The intuitive results were recorded with an Eclipse80i microscope (Nikon, Minato, Japan) and the quantification of the mineralized extracellular matrix stained by alizarin red S and collagen stained by alkaline phosphatase staining was detected by a multifunctional microplate reader (Varioskan Flash; Thermo Fisher Scientific, USA) at a wavelength of 405 nm.

Scratch assay**:** The effect of HAp, HL8, HL16, and HL24 scaffolds on HUVECs migration activity was studied using the scratch assay. Briefly, HUVECs were cultured in a six-well plate (Corning, New York, USA) for 12 hours to allow the cells to fully cover the bottom of the plate, and then the scratch was made with a 10 μL pipette head. Each well was filled with serum-free DMEM and 3 specimens were placed in the upper chamber of the corresponding well. After 24 hours of co-culture, the healing of the scratch area was observed under an optical microscope (LSCM, LSM800, Zeiss, Germany).

Transwell assay**:** Transwell plates with a membrane pore size of 8 μm (Corning, NY, USA) were involved to conduct the transwell assay, where each lower chamber was loaded with one specimen of HAp, HL8, HL16, or HL24 scaffolds and 500 μL serum-free DMEM. HUVECs were cultured on the upper chamber of the transwell and allowed for 24 hours migration. Cells migrated from the upper chamber to the bottom of each well were then immersed in 4% paraformaldehyde (Gibco, USA) for 30 min and stained with a 0.5% crystal violet solution for another 30 min. Finally, three randomly selected visual fields and corresponding images were taken and analyzed with an optical microscope (LSCM, LSM800, Zeiss, Germany).

Tube formation assay**:** To detect the tube formation ability of the HAp, HL8, HL16,

and HL24 scaffolds, Matrigel (BD BioCoat Matrigel, USA) was spread on the bottom of a 24-well plate and HUVECs were cultured in the same way as described in the section of in vitro biocompatibility evaluation. After 8-hour incubation, tube formation was observed under an optical microscope (LSCM, LSM800, Zeiss, Germany). Further quantitative analysis of the number of joint and total tube length was conducted with ImageJ software.

qRT-PCR analysis**:** MC3T3-E1 and HUVECs were cultured in the same way as described in the section of in vitro biocompatibility evaluation for 7 days. Trizol reagent (Life Technologies) and the RNeasy Mini kit (Qiagen, Valencia, CA) were used to extract the total RNA for the following concentration measurement with a NanoDrop spectrophotometer (Thermo Fisher Scientific, USA). The extracted RNA was subsequently transcribed to cDNA using an RNA-to-cDNA kit (Applied Biosystems, Foster City, USA). Next, Primer (version 5.0, Premier Biosoft International, USA) was used to design the primers with specific base sequences for target genes (Figure S19). The expression level of osteogenic and angiogenic genes was normalized by housekeeping gene GAPDH with the ΔΔcT method.

Western blot assay**:** The BCA protein kit (Beyotime, P0012, China) was used to detect the concentration of total protein from treated MC3T3-E1 and HUVECs. SDS-PAGE gel loading with 30 μg of protein was transferred onto a polyvinylidene difluoride (PVDF) membrane. Nonspecific bounding was then blocked with 5% dilute skim milk before the PVDF membrane was later incubated overnight with primary antibodies including Wnt1, Runx2, OCN, and VEGF. The corresponding secondary antibody was then incorporated with primary antibodies to make the target proteins be visualized under the chemiluminescence machine (ECL, Thermo Fisher Scientific, CA, USA). Bio-Rad image analysis software (Bio-Rad, Hercules, CA, USA) and ImageJ software were used to analyze the optical density of the bands and the concentration of the antigen−antibody complexes.

Transcriptome sequencing**:** The magnetic beads with Oligo (dT) were used to enrich the eukaryotic mRNA of treated MC3T3-E1. The enriched mRNA was then interrupted into short fragments with interrupting reagent for the following procedures.

One-strand cDNA was synthesized by six-base random primers while two-strand cDNA was prepared by a two-strand reaction system. Next, PCR amplification was conducted upon a specific fragment size after the purification and repair of the two-strand cDNA. Agilent 2100 Bioanalyzer was used to evaluate the constructed library and the Illumina sequencer was used for sequencing. The Ingenuity Pathway Analysis software (IPA®, v01-04, QIANGEN Redwood City, CA, USA) was used to construct the potential gene interaction network and involved signaling pathway.

Calvarial defect model and scaffold implantation**:** Calvarial defect models with four defects (6mm diameter) were established in New Zealand White Rabbits (Charles River, USA) anesthetized by intravenous injecting 3% pentobarbital (1 mL/kg). Each defect was sterilized and then implanted with HAp, HL8, HL16, and HL24 scaffolds in corresponding positions. All surgical experiments strictly abided by the National Institutes of Health Guidelines and the procedures were approved by the Animal Care and Use Committee of Peking Union Medical College Hospital. Penicillin (Shengwang Corp, Shandong, China) was intramuscularly injected at 0, 24, and 48 hours to prevent postoperative infection.

Micro-CT scanning and analysis: The New Zealand White Rabbits were sacrificed at 0, 1, and 2 months after surgery to harvest the calvarial bone. The harvested calvarial bone tissue was fixed by 4% paraformaldehyde solution at room temperature for 2 days. A Micro-computed tomography machine (Micro-CT; Siemens, Germany) was used to take the horizontal, coronal, and 3D reconstructed Micro-CT scanning images to evaluate the new bone formation. Mimics software (Materialise, Leuven, Belgium) was used to quantitively analyze the bone volume and the bone mineral density (BMD) of the newly formed bone tissue.

Immunofluorescence staining**:** The decalcified calvarial bone was immersed into formalin for 24 h fixation, followed by a histologic section. The immunofluorescence staining of the sectioned calvarial bone tissue was then performed with specific antibodies (Laminin, CD31) according to the procedure.

Chicken chorioallantoic membrane (CAM) assay: Fertilized chicken eggs on the 8th day were used to conduct the CAM assay. Specifically, the CAM was exposed by

drilling a 1.0 cm diameter hole on the eggshell and the HAp, HL8, HL16, and HL24 scaffolds were placed on the CAM for 5 days under a condition of 37.8 °C and 80% humidity to induce vessel generation. Gross photographs of CAM neovascularization were taken and the quantitative analyses were performed using ImageJ software.

Histological evaluation**:** The calvarial bone was harvested and decalcified in ethylenediaminetetraacetic acid (EDTA) solution (10%) for 2 months with a refresh of the EDTA every 3 days. After decalcification, the calvarial bone was embedded into the paraffin and sliced into sections with a thickness of 5 μm. H&E and Masson's trichrome staining were conducted to qualitatively evaluate new bone formation level with an optical microscope (LSCM, LSM800, Zeiss, Germany).

Statistical Analysis**:** Data are expressed as mean ± standard deviation (S.D). Statistical comparisons were made by one-way ANOVA (for multiple comparisons): $^{*}p < 0.05$, $^{**}p < 0.01$, $^{***}p < 0.001$.

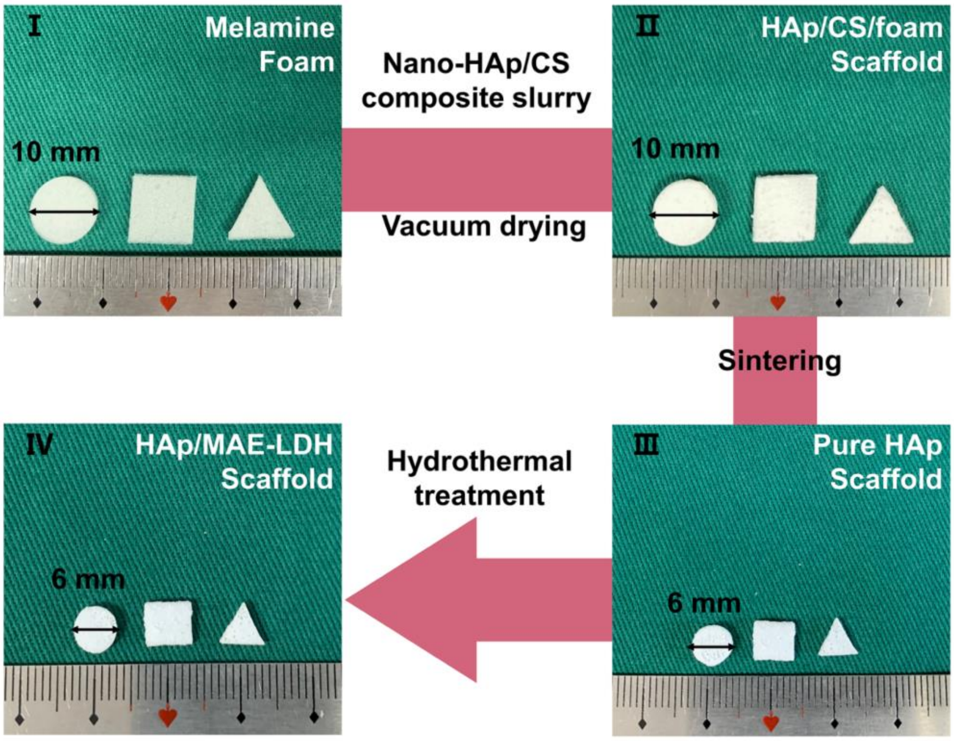

Figure S1. The procedure for preparation of HAp/MAE-LDH scaffolds.

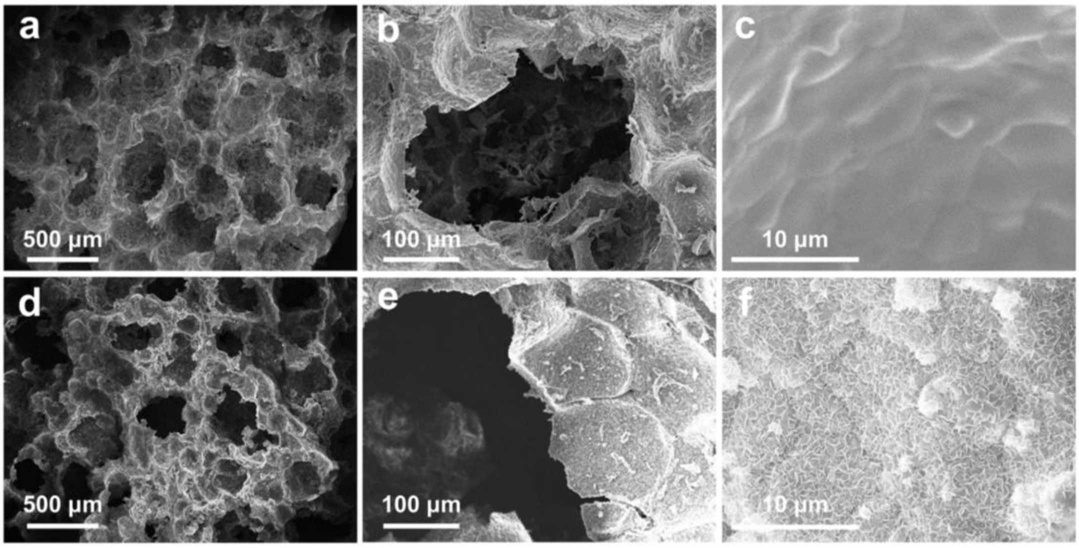

Figure S2. SEM images of (a, b, c) HAp and (d, e, f) HAp/MAE-LDH scaffolds.

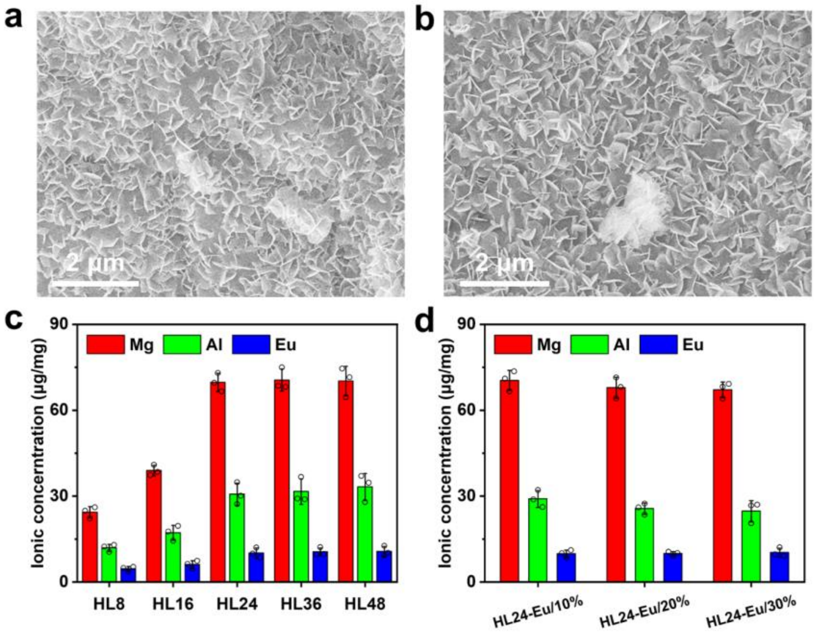

Figure S3. SEM morphologies of (a) HL36 and (b) HL48 scaffolds. (c) The elemental contents of HL8, HL16, HL24, HL36 and HL48 scaffolds. (d) The elemental contents of HL24-Eu/10%, HL24-Eu/20% and HL24-Eu/30% scaffolds. Data are presented as mean values ± s.d. (n = 3).

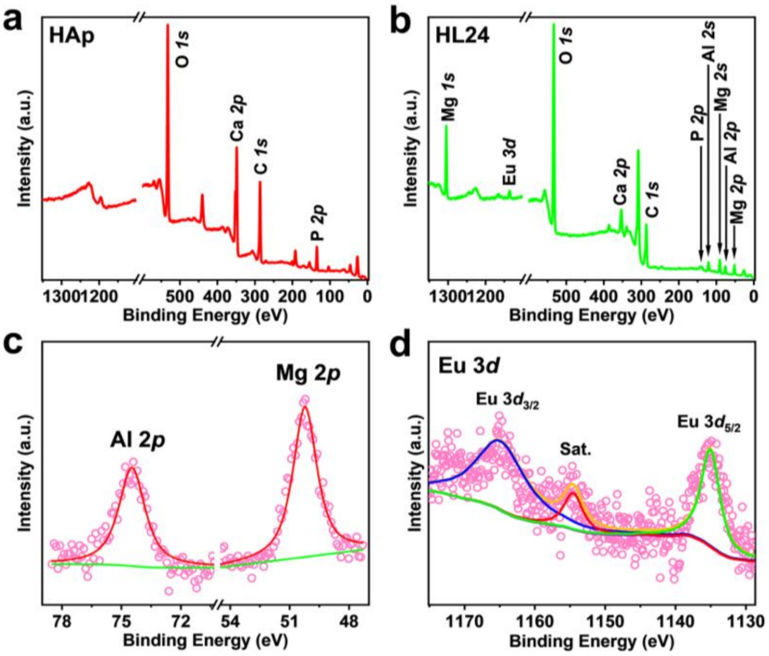

Figure S4. XPS full spectra of (a) HAp and (b) HL24 scaffolds. (c) The Mg 2p, Al 2p and (d) Eu 3d spectra of HL24 scaffold.

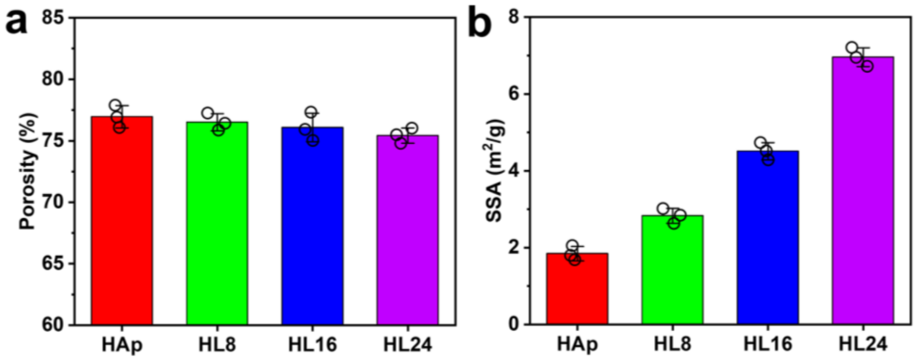

Figure S5. (a) Porosity and (b) specific surface area of HAp, HL8, HL16 and HL24 scaffolds. Data are presented as mean values ± s.d. (n = 3).

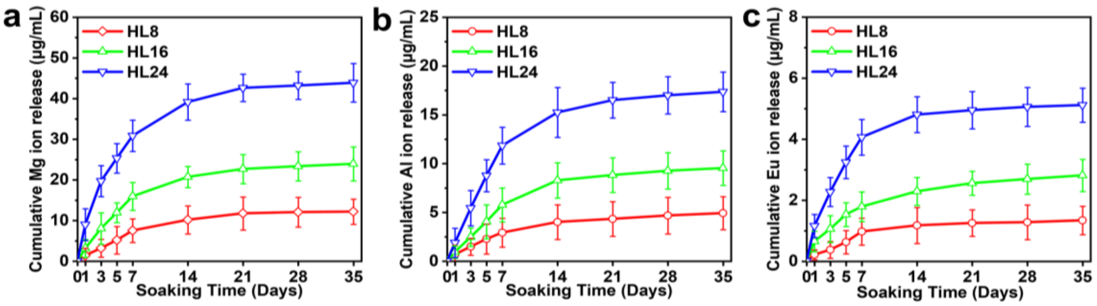

Figure S6. (a) The Mg, (b) Al, and (c) Eu ions release kinetics of HL8, HL16 and HL24 scaffolds, respectively. Data are presented as mean values ± s.d. (n = 3).

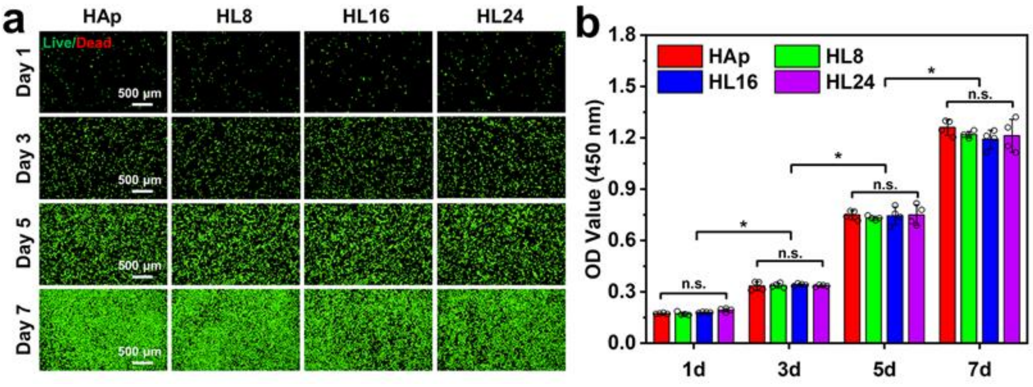

Figure S7. (a) Calcein-AM/PI (live/dead) staining and (b) CCK-8 assay results evaluating the biocompatibility of HAp, HL8, HL16, and HL24 scaffolds for MC3T3-E1 up to the 7th day. Data are presented as mean values ± s.d. (n = 4). $*p < 0.05$ (one-way ANOVA).

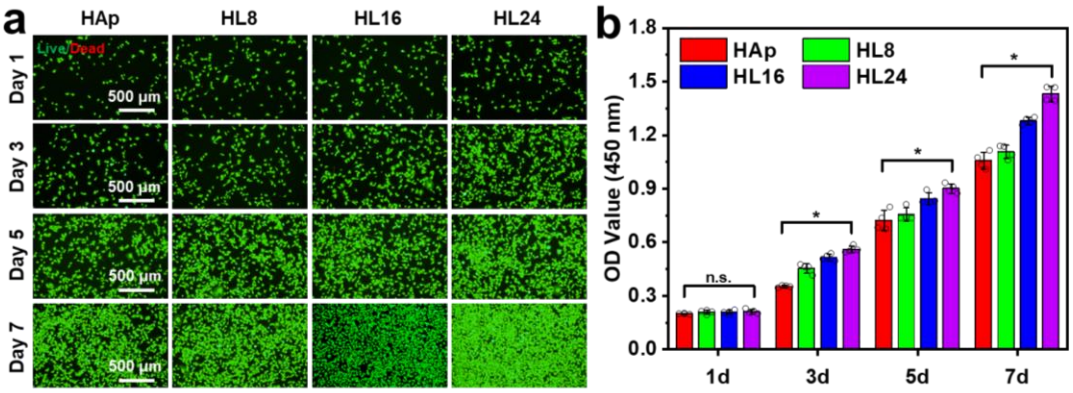

Figure S8. (a) Calcein-AM/PI (live/dead) staining and (b) CCK-8 assay results evaluating the biocompatibility of HAp, HL8, HL16, and HL24 scaffolds for HUVECs up to the 7th day. Data are presented as mean values ± s.d. (*n* = 4). $*p < 0.05$, $**p < 0.01$ (one-way ANOVA).

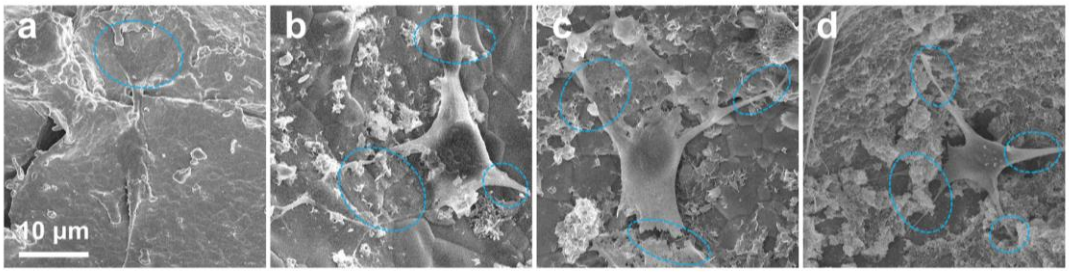

Figure S9. SEM images of MC3T3-E1 cells cultured on (a) HAp, (b) HL8, (c) HL16 and (d) HL24 scaffolds for 3 days.

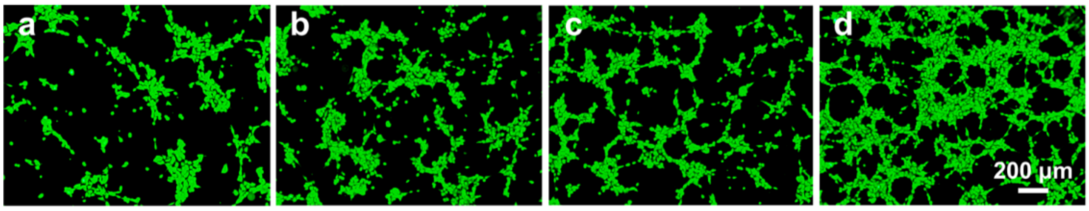

Figure S10. Matrigel experiment results accessing the vessel formation capability of HUVECs cultured with (a) HAp, (b) HL8, (c) HL16, and (d) HL24 scaffolds.

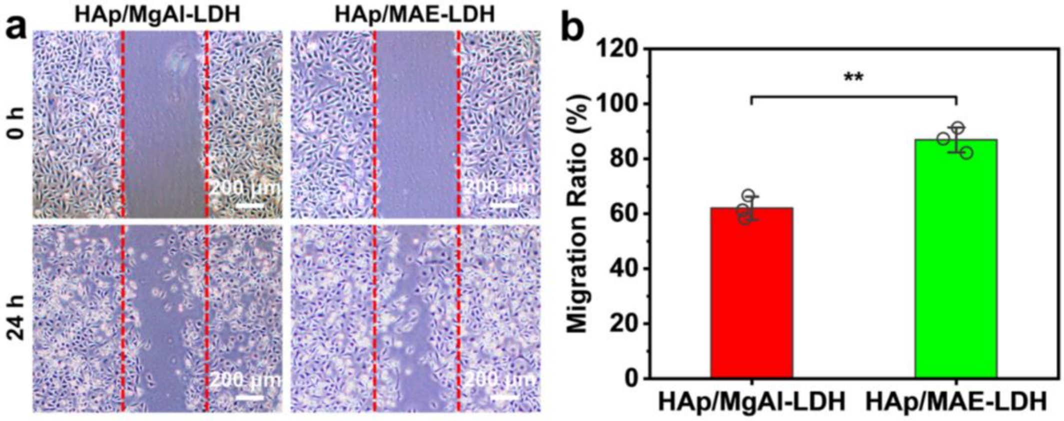

Figure S11. Optical microscope images and further quantitative analysis results (ImageJ 1.52q1.52v software) of scratch assay evaluating the migration activity of HUVECs cultured with HAp/MgAl-LDH or HAp/MAE-LDH. Data are presented as mean values ± s.d. (n = 3). **p < 0.01 (one-way ANOVA).

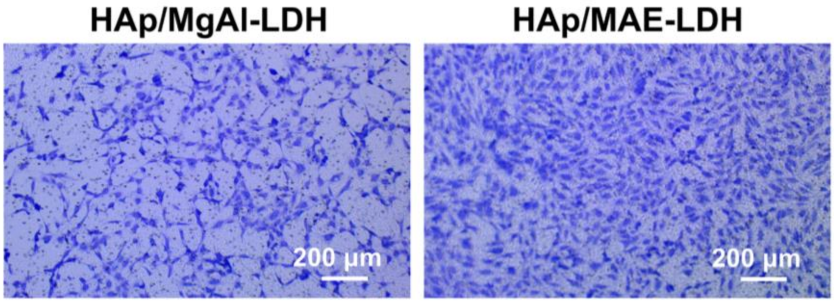

Figure S12. Migrated HUVECs cultured with HAp/MgAl-LDH or HAp/MAE-LDH stained with crystal violet in Transwell assay.

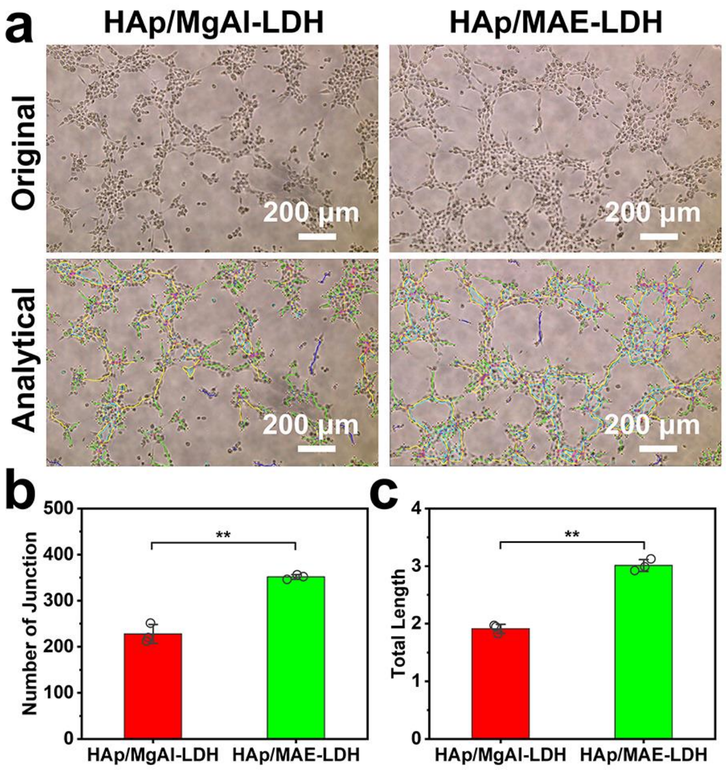

Figure S13. Matrigel experiment results and further quantitative analysis results (ImageJ 1.52q1.52v software) accessing the vessel generation capability of HUVECs cultured with HAp/MgAl-LDH or HAp/MAE-LDH. Data are presented as mean values ± s.d. (n = 3). **p < 0.01 (one-way ANOVA).

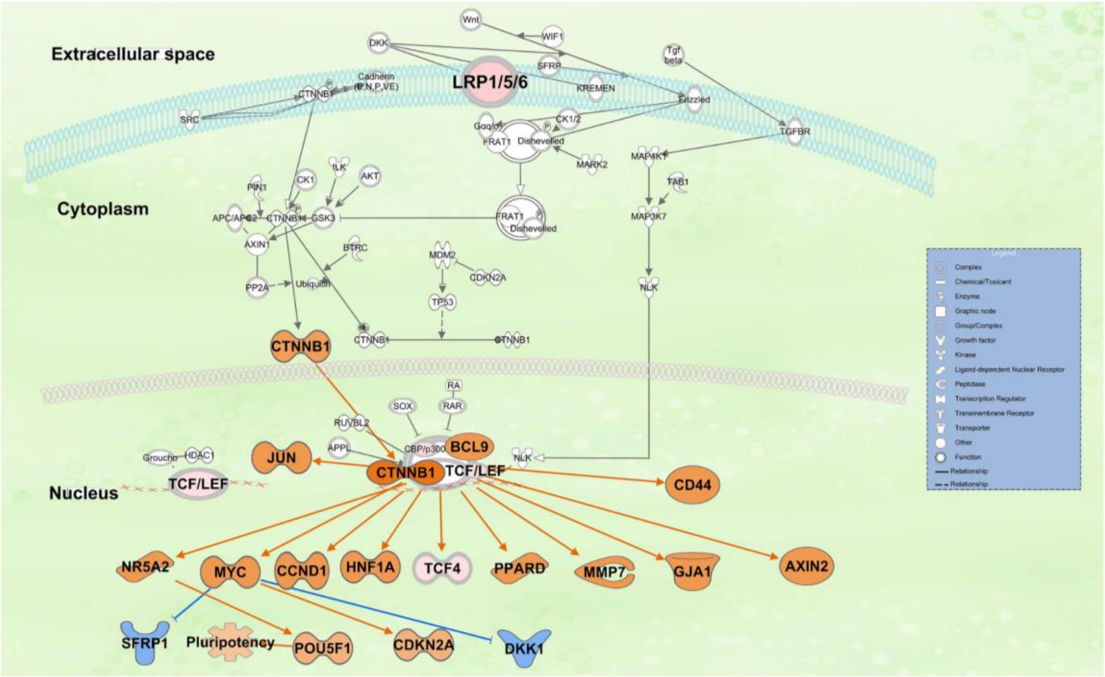

Figure S14. The differentially expressed genes clustered in the wnt/β-catenin signaling pathway.

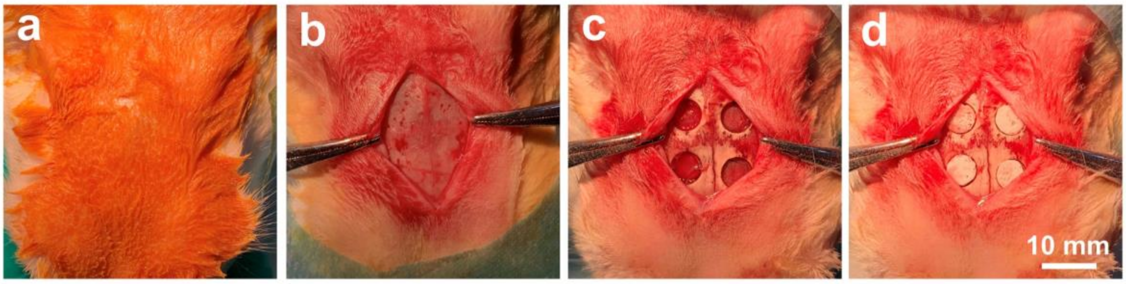

Figure S15. The skull defect model of New Zealand White Rabbits treated by (a) HAp, (b) HL8, (c) HL16, and (d) HL24 scaffolds.

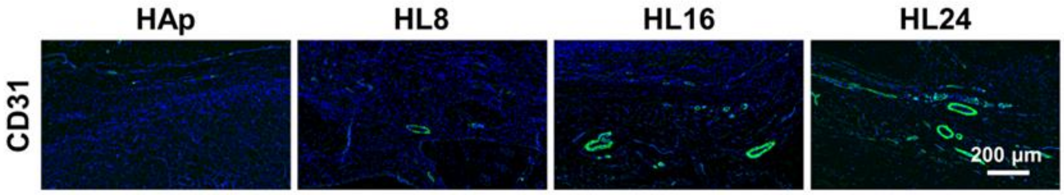

Figure S16. Regenerated vessels marked by CD31 induced by HAp, HL8, HL16, and HL24 scaffolds.

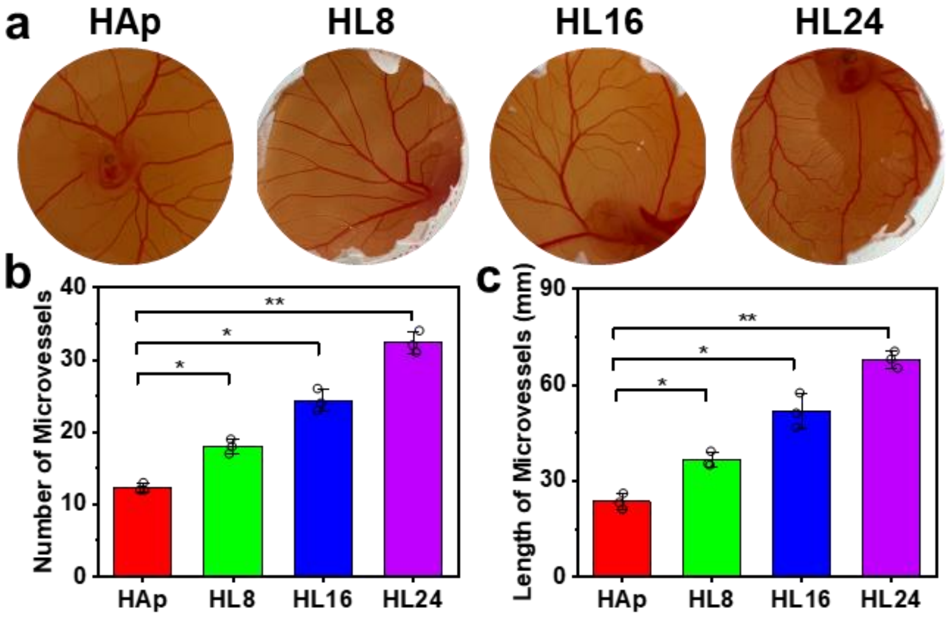

Figure S17. Gross photographs and further quantitative analyses of neovascularization induced by HAp, HL8, HL16, and HL24 scaffolds of the chicken chorioallantoic membrane assay.

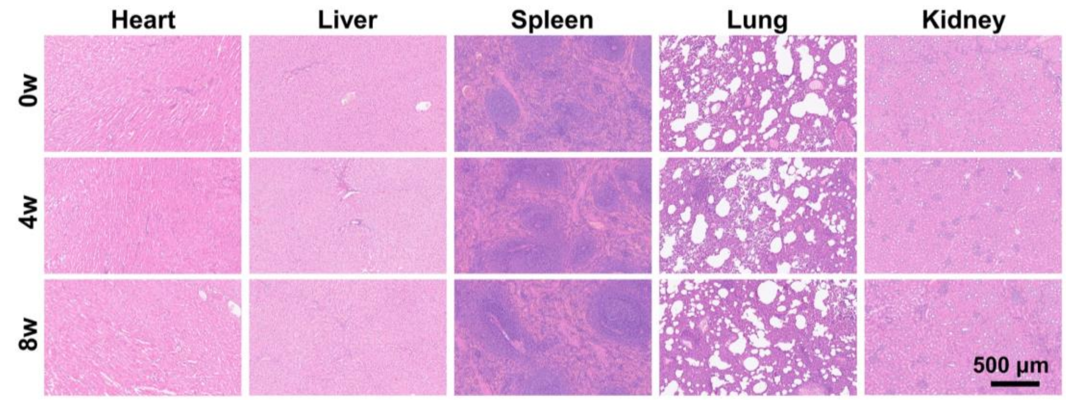

Figure S18. The heart, liver, spleen, lung, and kidney tissue were taken and sliced at 0-week, 4-week and 8-week after HL24 implantation to evaluate the systemic toxicity of the HL24 scaffolds.

Gene Name	Forward Sequences	Reverse Sequences
GAPDH Mouse	ATGCCAGTGAGCTTCCCGTTCAG	CATCACTGCCACCCAGAAGACTG
GAPDH Human	ACCACCCTGTTGCTGTAGCCAA	GTCTCCTCTGACTTCAACAGCG
OCN Mouse	CTCCTGAAAGCCGATGTGGTCA	CGCTACCTGTATCAATGGCTGG
RunX2 Mouse	TCATCTGGCTCAGATAGGAGGG	CCTGAACTCTGCACCAAGTCCT
Wnt1 Human	CGATGGAACCTTCTGAGCAGGA	CTCTTCGGCAAGATCGTCAACC
Wnt1 Mouse	GATGAACGCTGTTTCTCGGCAG	CGAGAGTGCAAATGGCAATTCCG
VEGF Human	GATGGCAGTAGCTGCGCTGATA	TTGCCTTGCTGCTCTACCTCCA

Figure S19. Gene primer sequences of GAPDH, OCN, Wnt1, RunX2, and VEGF used in qPCR experiments.

References

[1] K. Zhou, P. Yu, X. Shi, T. Ling, W. Zeng, A. Chen, W. Yang, Z. Zhou, *ACS Nano* **2019**, *13*, 9595.

[2] M. Chen, Y. Li, S. Liu, Z. Feng, H. Wang, D. Yang, W. Guo, Z. Yuan, S. Gao, Y. Zhang, K. Zha, B. Huang, F. Wei, X. Sang, Q. Tian, X. Yang, X. sui, Y. Zhou, Y. Zheng, Q. Guo, *Bioact. Mater.* **2021**, *6*, 1932.
